# Supplementary material for: Efficient curation of genebanks using next generation sequencing reveals substantial duplication of germplasm accessions
Source: Sci Rep. 2019 Jan 24;9:650. doi: 10.1038/s41598-018-37269-0 (PMC6346010; doi:10.1038/s41598-018-37269-0)

**Supplementary information**

**Article title**: Efficient Curation of Genebanks Using Next Generation Sequencing Reveals Substantial Duplication of Germplasm Accessions

**Authors**: Narinder Singh, Shuangye Wu, W. John Raupp, Sunish Sehgal, Sanu Arora, Vijay Tiwari, Prashant Vikram, Sukhwinder Singh, Parveen Chhuneja, Bikram S. Gill and Jesse Poland

The following Supporting Information is available for this article:

**Figure S1**. Plots showing per sample distribution of (A) barcoded read counts, (B) 64-mer unique tags, (C) percent heterozygosity per sample, and (D) percent missing data per SNP.

**Figure S2**. Cluster analysis of WGRC, PAU and CIMMYT genebanks' *Aegilops tauschii* collection using genotyping-by-sequencing.

**Figure S3**. Percent identity by state (pIBS) distributions for (A) all genebanks collectively, (B) WGRC genebank, (C) CIMMYT genebank, and (D) PAU genebank.

**Figure S4**. Bar plot showing frequency of each group size.

**Figure S5**. Virtual gel image showing accessions from four different groups (lanes 1 and 5-9 from Grp190, lane 2 from Grp476, lanes 3-4 from Grp523 and lane 10 from Grp529).

**Figure S6**. Virtual gel image showing accessions from four different groups (lanes 1, 3, and 4 are from Grp188; lane 2 from Grp187; lanes 5-7 from Grp15; and lanes 8-10 from Grp37).

**Figure S7**. Virtual gel image showing gliadin profiling for heterogeneous accession TA1714.

**Figure S8**. Virtual gel image showing gliadin profiling for homogeneous accessions TA2457.

**Table S1**. List of *Ae. tauschii* accessions from different genebanks.

**Table S2**. List of matching and unique *Ae. tauschii* accessions.

**Table S3**. Imputed posterior probabilities for each accession with missing passport data.

Figure S1. Plots showing per sample distribution of (A) barcoded read counts, (B) 64-mer unique tags, (C) percent heterozygosity per sample, and (D) percent missing data per SNP.

**(A)**

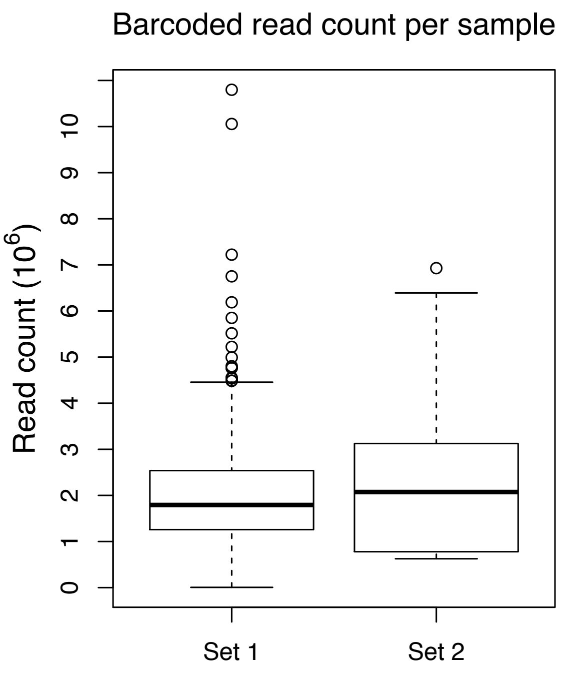


**(B)**

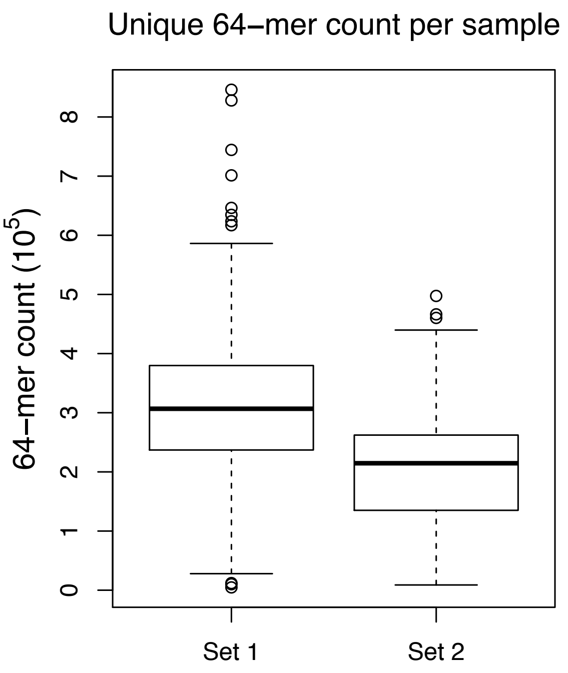


**(C)**

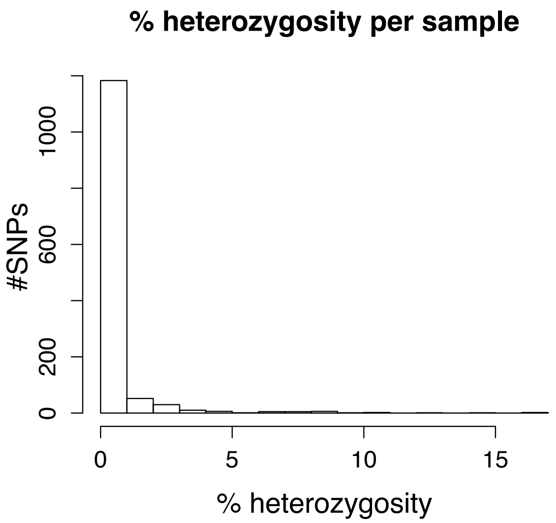


**(D)**

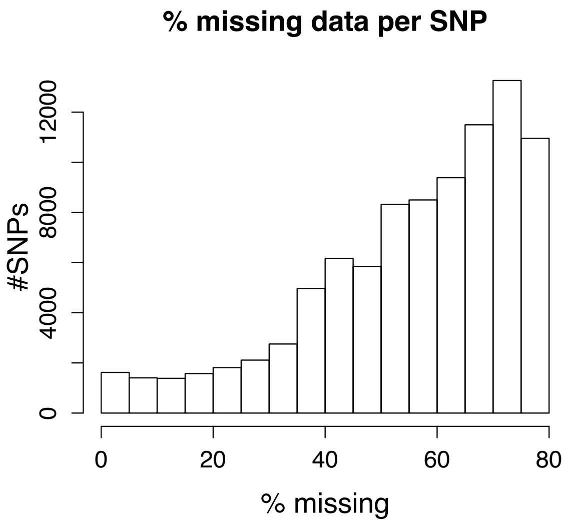

Figure S2. Cluster analysis of WGRC, PAU and CIMMYT genebanks' *Aegilops tauschii* collection using genotyping-by-sequencing.

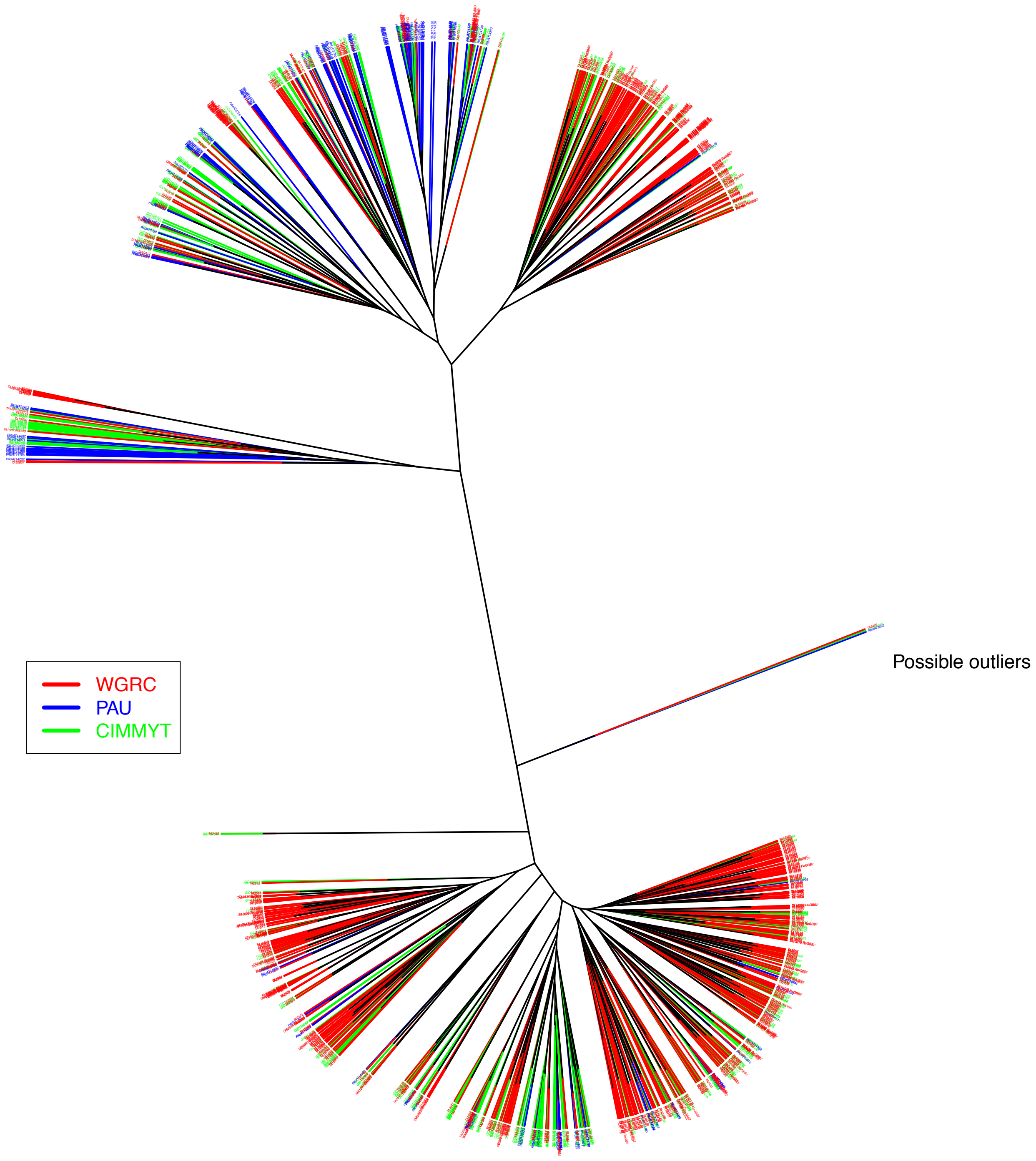

Figure S3. Percent identity by state (pIBS) distributions for (A) all genebanks collectively, (B) WGRC genebank, (C) CIMMYT genebank, and (D) PAU genebank.

**(A)**

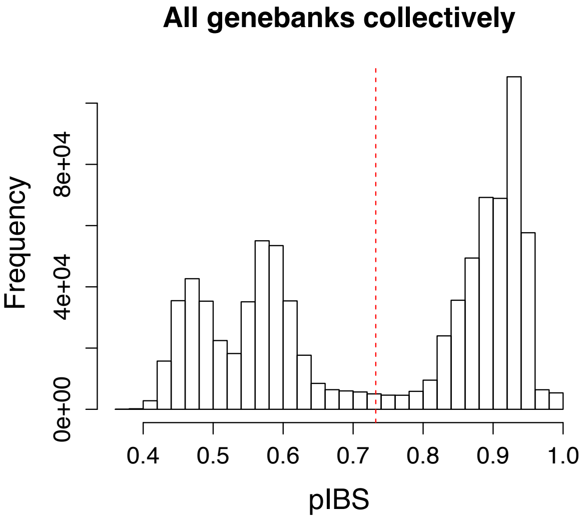


**(B)**

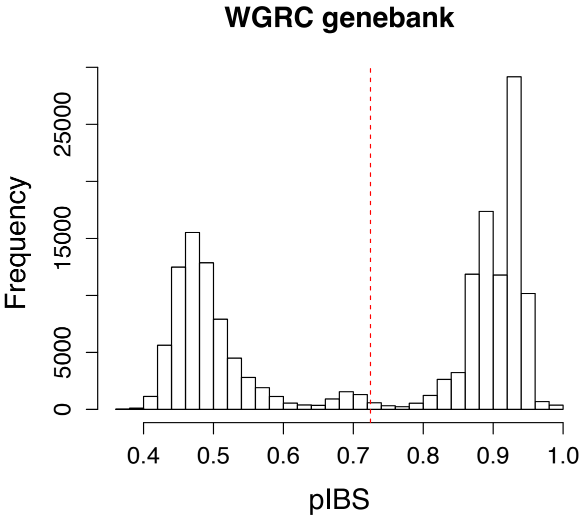


**(C)**

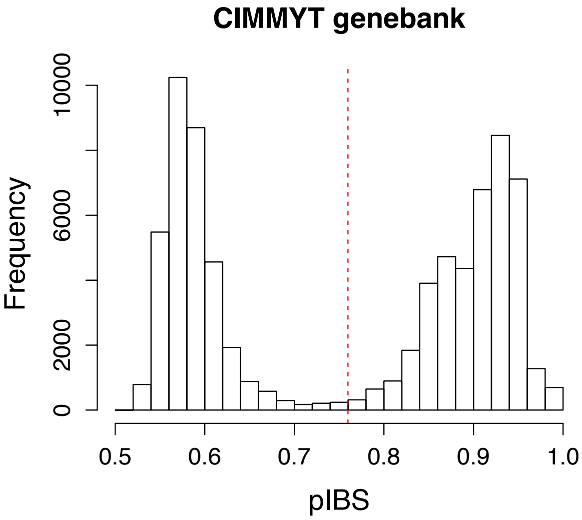


**(D)**

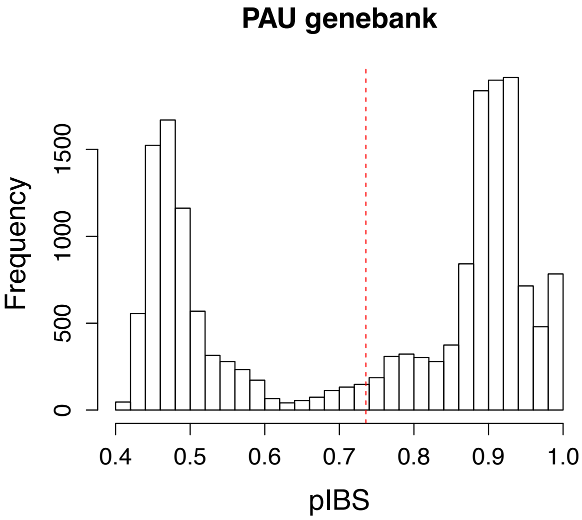

Figure S4. Bar plot showing frequency of each group size. Values on top of each bar represents the exact frequency of corresponding group size listed on x-axis. Total of 368 accessions were total unique and did not match with any other accession.

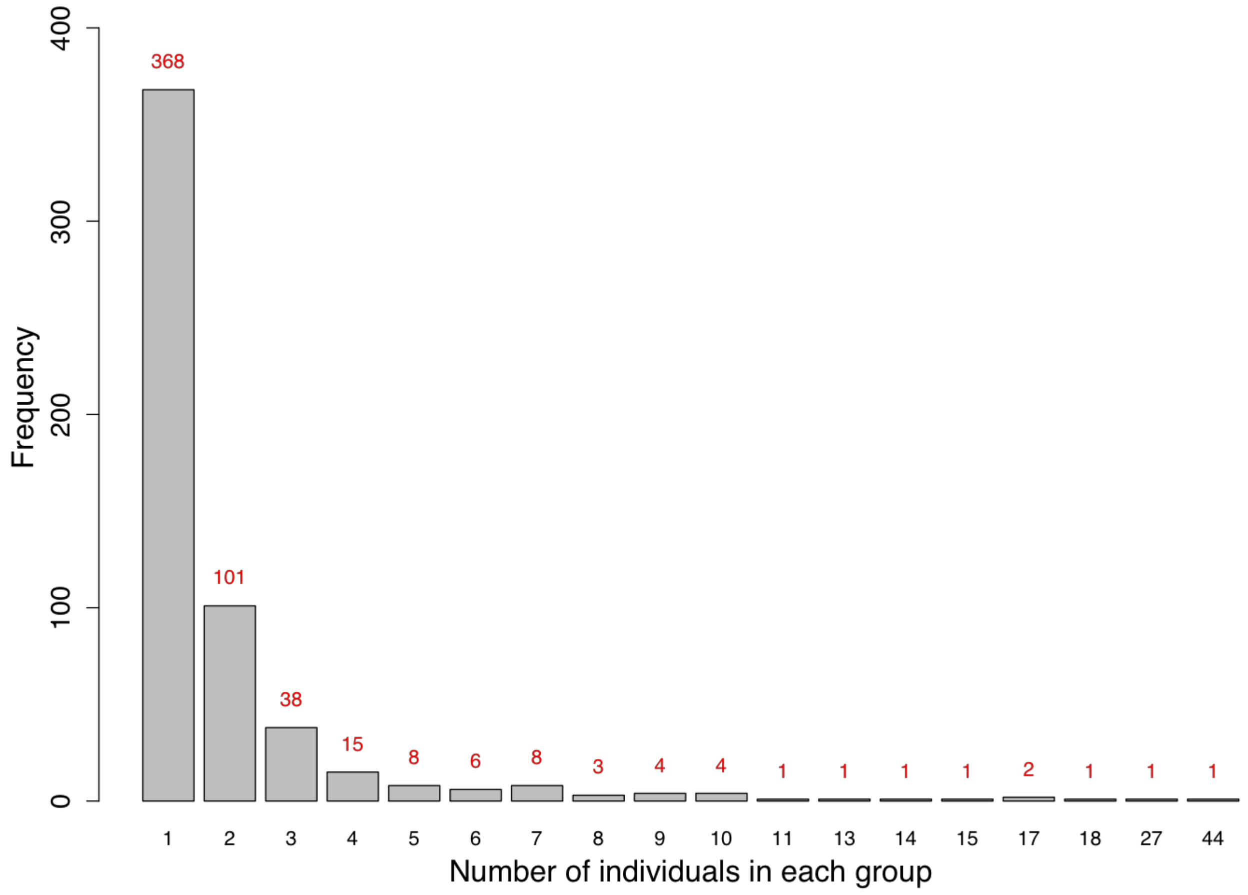

Figure S5. Virtual gel image showing accessions from four different groups (lanes 1 and 5-9 from Grp190, lane 2 from Grp476, lanes 3-4 from Grp523 and lane 10 from Grp529). As expected, lanes 2 and 10 shows different banding pattern as they are the only representative of their respective groups on this gel. Lanes 3 and 4 have similar banding pattern. Lanes 1 and 5-9 from Grp190 have similar banding pattern. This suggests that accessions within a group tend to have a similar banding pattern, which corroborates with the accession grouping with allele matching.

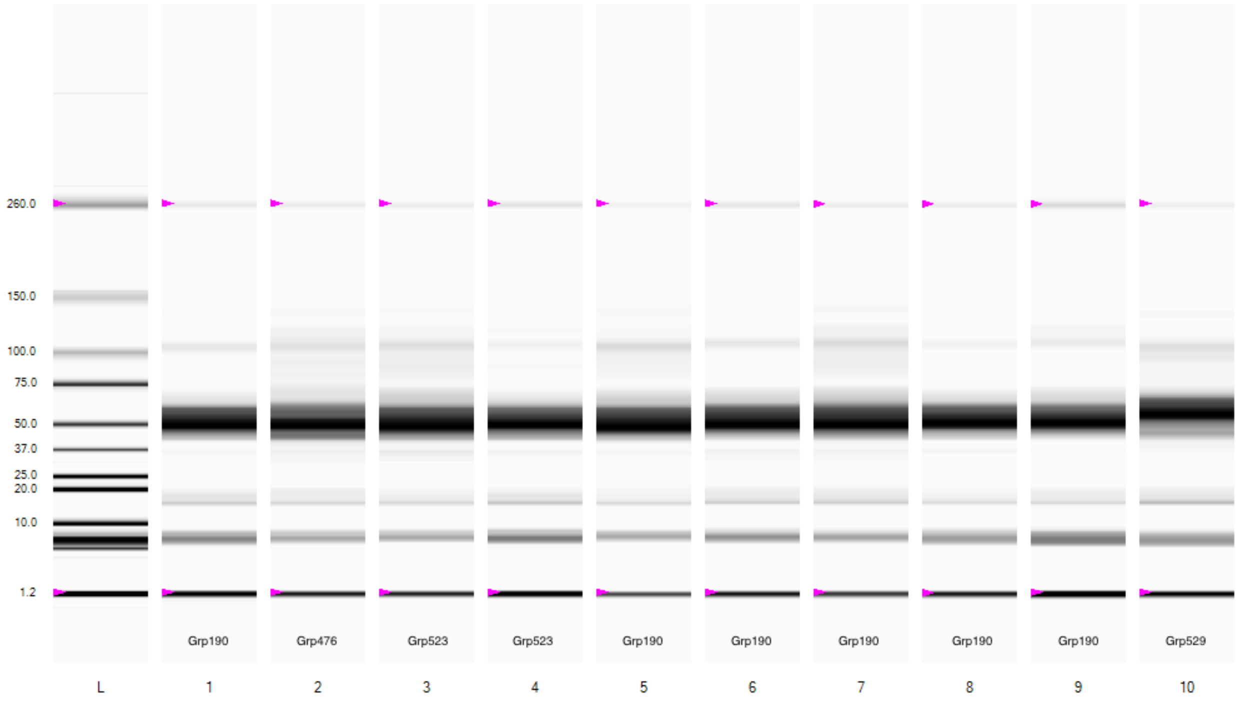

Figure S6. Virtual gel image showing accessions from four different groups (lanes 1, 3, and 4 are from Grp188; lane 2 from Grp187; lanes 5-7 from Grp15; and lanes 8-10 from Grp37). Lanes 1,3 and 4 have similar banding pattern; lane 2 has totally different banding pattern not matching with any other lane; lanes 5-7 have similar banding pattern but lane 7 (green arrow) seems to have very high concentration of the protein, giving it a smear look; lanes 8-10 seem to have similar banding pattern. This suggests that accessions within a group tend to have a similar banding pattern, which corroborates with the accession grouping with allele matching.

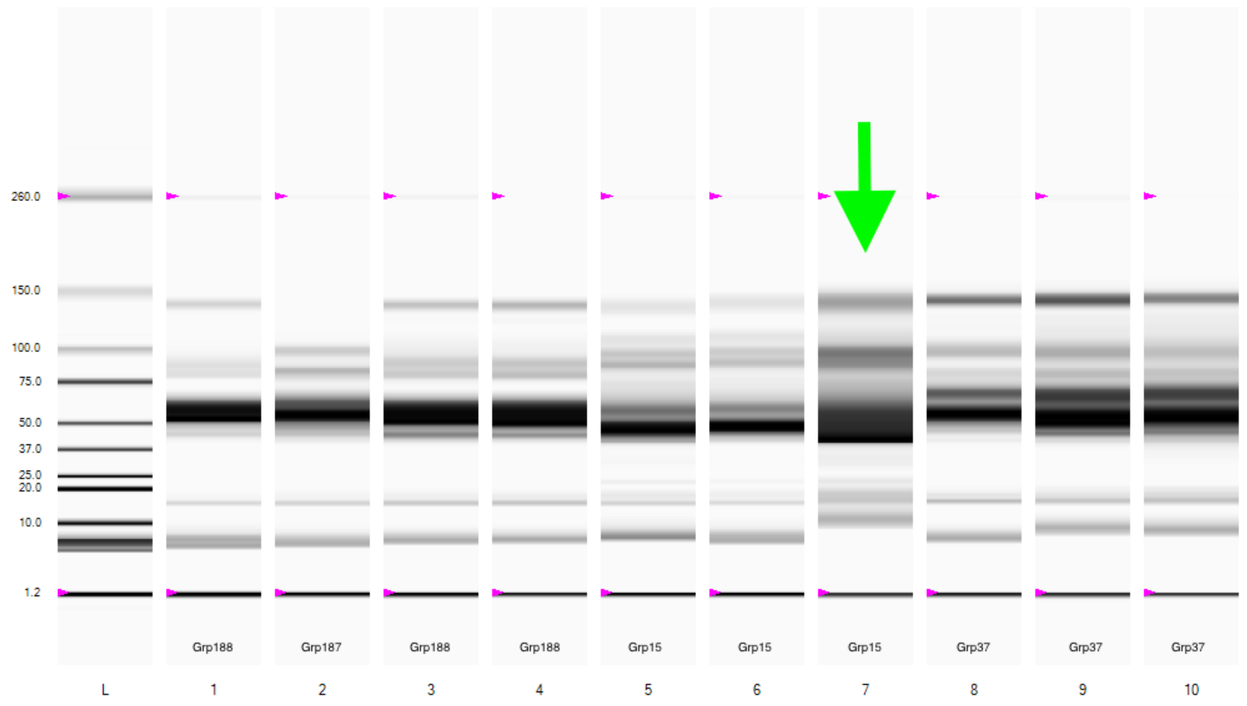

Figure S7. Virtual gel image showing gliadin profiling for heterogeneous accession TA1714. First two lanes (red box) have a similar banding pattern forming a group, and lanes 3-9 (blue box) have similar banding pattern with minor differences. Lane 10 is Chinese spring wheat for control. The different patterns between red and blue box samples presents an evidence that the samples came from a heterogeneous seed source.

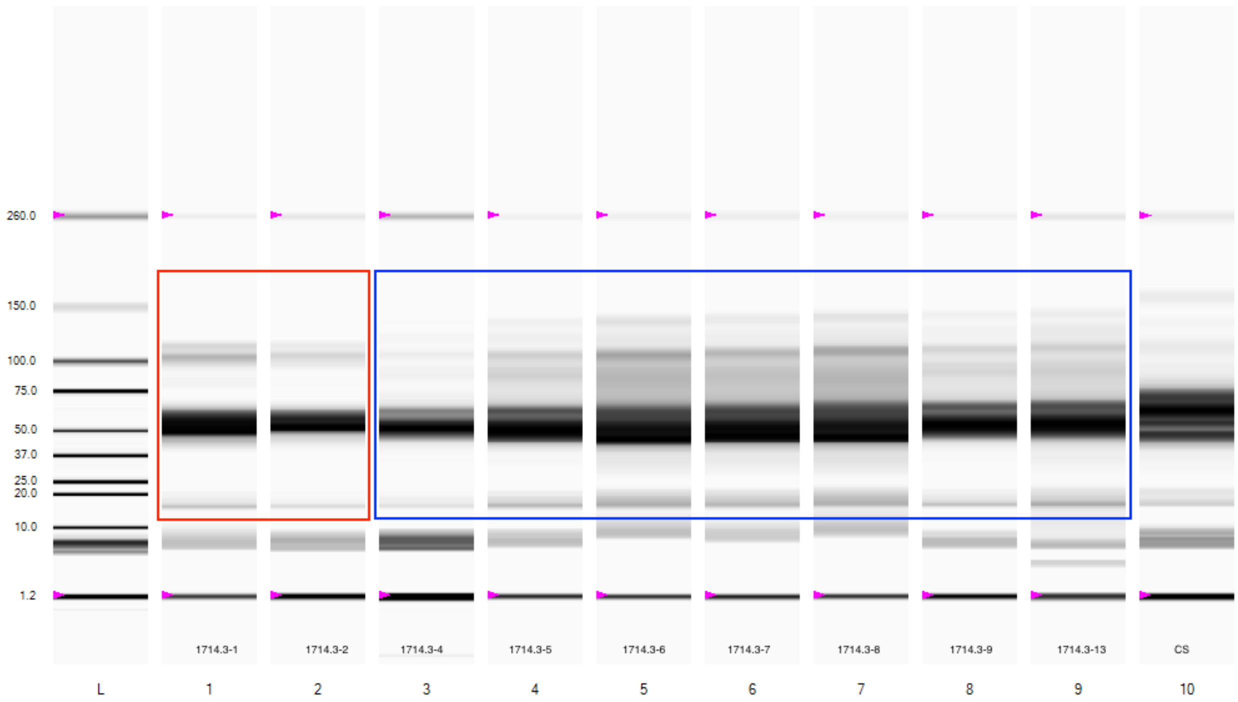

Figure S8. Virtual gel image showing gliadin profiling for homogeneous accessions TA2457. With some minor differences, banding pattern for lanes 1-9 (green box) look similar with an exception of lane 8 (green arrow). Sample in lane 8 does appear to have a similar banding pattern but possibly has higher extracted protein concentration that gives it a smeared look.

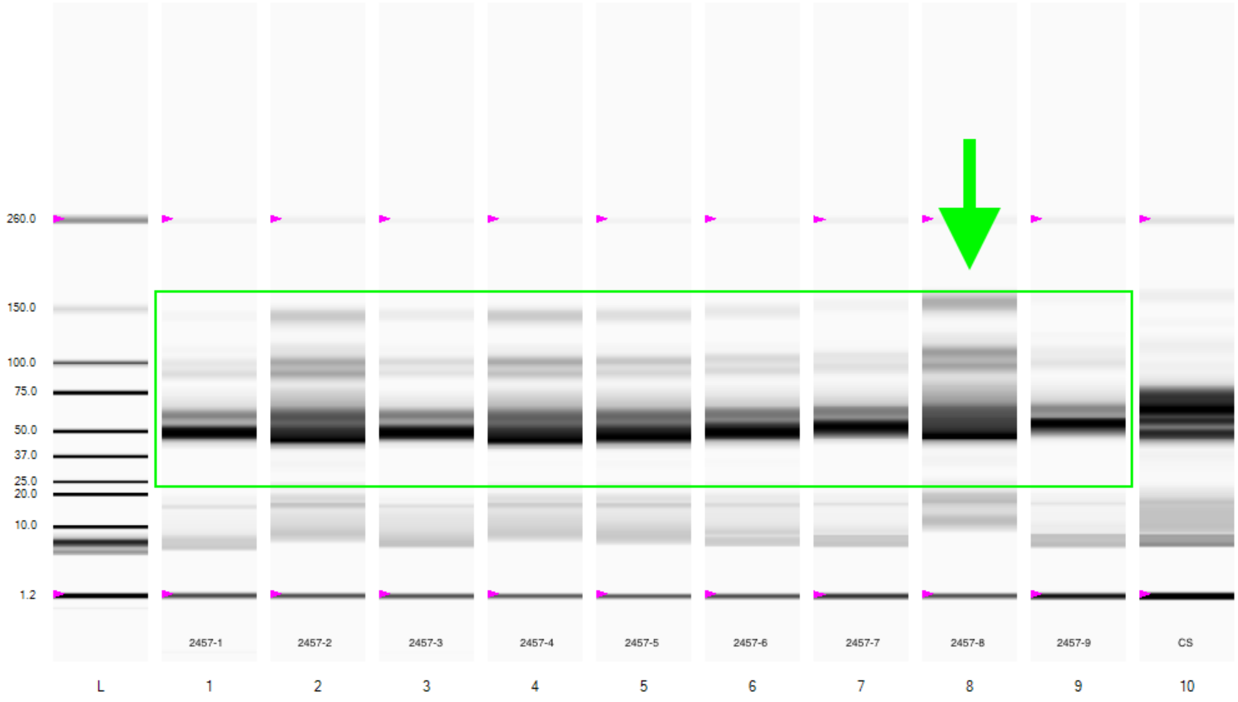

Supplement: Supplementary file 1 — Supplemental Information [file 41598_2018_37269_MOESM1_ESM.pdf]
